# Constraint-Induced Movement Therapy Combined with Transcranial Direct Current Stimulation over Premotor Cortex Improves Motor Function in Severe Stroke: A Pilot Randomized Controlled Trial

**DOI:** 10.1155/2017/6842549

**Published:** 2017-01-30

**Authors:** Suellen M. Andrade, Larissa M. Batista, Lídia L. R. F. Nogueira, Eliane A. de Oliveira, Antonio G. C. de Carvalho, Soriano S. Lima, Jordânia R. M. Santana, Emerson C. C. de Lima, Bernardino Fernández-Calvo

**Affiliations:** Federal University of Paraíba, João Pessoa, PB, Brazil

## Abstract

*Objective.* We compared the effects of transcranial direct current stimulation at different cortical sites (premotor and motor primary cortex) combined with constraint-induced movement therapy for treatment of stroke patients.* Design.* Sixty patients were randomly distributed into 3 groups: Group A, anodal stimulation on premotor cortex and constraint-induced movement therapy; Group B, anodal stimulation on primary motor cortex and constraint-induced movement therapy; Group C, sham stimulation and constraint-induced movement therapy. Evaluations involved analysis of functional independence, motor recovery, spasticity, gross motor function, and muscle strength.* Results.* A significant improvement in primary outcome (functional independence) after treatment in the premotor group followed by primary motor group and sham group was observed. The same pattern of improvement was highlighted among all secondary outcome measures regarding the superior performance of the premotor group over primary motor and sham groups.* Conclusions.* Premotor cortex can contribute to motor function in patients with severe functional disabilities in early stages of stroke. This study was registered in ClinicalTrials.gov database (NCT 02628561).

## 1. Introduction

Stroke can sometimes cause severe disability and despite recent progress in the development of traditional rehabilitation, recovery of motor function is often incomplete, leading to the need for other resources as alternative treatment [1, 2]. In this sense, transcranial direct current stimulation (tDCS) has shown promising results in the sensorimotor and cognition areas, representing a potential tool for enhancing motor learning [3].

The possibility of combining physical therapy modalities is one of the advantages of tDCS in the rehabilitation process. Higher gains have been demonstrated when tDCS is applied in association with physical rehabilitation than if the exercises were performed alone [4, 5]. In a recent study, a group of researchers linked to the Cochrane Collaboration analyzed systematic reviews related to interventions involving improvement of upper limb function after stroke [6]. The researchers found that there is evidence of moderate quality which suggests that constraint-induced movement therapy (CIMT) is an effective therapeutic modality for the treatment of patients with hemiparesis. Thus, we chose CIMT as a protocol for physical rehabilitation of this study, since previous studies have already demonstrated that this rehabilitation program was able to promote alterations in sensory-motor cortical activation and corticospinal conductivity in patients after stroke [7], in addition to promoting significant clinical improvement [8]. Objectively, the substantially reduced use of the uninjured upper limb would decrease the activity of the contralesional motor cortex, which, in turn, could contribute to a reduction in the transcallosal inhibition of this region to the homologous area in the affected hemisphere. As a consequence, this could attenuate the interhemispheric imbalance, facilitating ipsilesional activation and increasing adaptive neural plasticity [9]. There are references that the neurophysiological changes are correlated to the behavioral gains after application of tDCS and CIMT in hemiparetic patients [10]. Plow et al. [11] point out that the CIMT plus tDCS can facilitate cortical activity and restore interhemispheric balance, representing an important adjuvant therapy for functional recovery.

Regarding the stimulation locus, most studies with poststroke tDCS involve stimulation of the primary motor cortex (M1) [12, 13]. In these studies, anodic stimulation provided greater clinical benefit than placebo stimulation [14, 15]. However, other researchers have postulated a conflicting point of view, stating that M1 does not present relevant clinical efficacy [16–18]. This inconsistency is most evident in studies involving patients with severe disability [19, 20]. There is evidence that more severe patients present cortical plasticity in regions other than M1, and therefore the involvement of other neural targets is important for the establishment of effective treatment strategies [21, 22]. In addition, studies show that much of the motor recovery occurs within three months after injury [23], which shows the importance of developing studies in early stages of stroke.

Therefore, other brain regions can be associated with complex motor performances and motor learning, including nonprimary motor areas, such as premotor cortex (PMC) [24]. Some main reasons may justify the choice for the PMC as a locus of stimulation: (a) PMC has a high survival rate after stroke. This fact is probably due to its great extension, since it occupies more than 60% of the frontal cortex [25]. In addition, upper motor areas are less affected by stroke involving the middle cerebral artery (the type of stroke with the highest incidence rate), while M1 is the most affected by lesions in the territory of this artery [26]; (b) PMC can facilitate functional recovery. Indeed, Craciunas et al. [27] found that brain metabolites related to neuronal and glial compartments are altered in the representation of the hands in bilateral motor and premotor areas and correlate to motor impairment of the distal and proximal arm after stroke. As PMC contributes with a high percentage of the spinal cortical tract (CST) to the hand [28], PMC could represent a useful path for upper limb recovery. A recent study has shown that increasing PMC excitability through anodic tDCS during observation of a motion sequence facilitates motor learning in healthy subjects. The authors suggest that PMC is a critical part of the neural circuitry related to motor memory, which is the elaborated by physical practice [29]; (c) PMC can affect the adaptive plasticity process. Dum and Strick [30] have shown that the PMC receives extensive contribution from the frontoparietal cortex and is directly associated with the generation and control of hand movements, independently of M1. Since there would be no clear hierarchical organization between M1 and PMC, our traditional view that M1 would play a higher role in relation to PMC would be seriously threatened. In addition, Marconi et al. [31] pointed to connections with homologous and heterologous contralesional cortices, showing a recruitment of both undamaged contralateral areas as well as compromised ipsilateral motor regions, reflecting an adaptive recovery strategy mediated by PMC.

Given the potential adjuvant effect of neurostimulation, our main objective is to verify whether active stimulation could promote additional gains to the CIMT results on the motor function of severely impacted postsubacute stroke patients. Our secondary objective is to investigate whether the stimulation of these two regions results in distinct clinical effects for the patients involved. In view of the reasons that point to the PMC as an alternative locus for treatment, we hypothesized that the application of tDCS on PMC is superior to M1 stimulation in severely affected patients after stroke. To our knowledge, this is the first study to compare efficacy of PMC tDCS versus M1 tDCS in stroke.

## 2. Methods

### 2.1. Design

This is a prospective, double blind, randomized, pilot study involving patients after stroke, undergoing stimulation sessions of transcranial direct current, and CIMT protocol. The protocol research was approved by the local Ethics Committee and conformed to the Declaration of Helsinki. All participants provided written informed consent for the experimental procedure. This study was conducted in a university laboratory from January to April 2016.

### 2.2. Participants

Sixty patients were recruited from the Stroke Unit of our department according to the following eligibility criteria: (a) age between 18 and 65 years; (b) diagnosis of unilateral, nonrecurring, subacute stroke, as defined by the International Classification of Diseases (ICD, 10) through Computed Tomography or Magnetic Resonance conducted by neurologists. Subacute stage was considered as an elapsed time of 01–03 months after vascular injury. Participants also had to be able, by using any method of pinch, to grasp a washcloth from a table top, lift it up a few inches, and release it. Because this protocol focused on the application of CIMT and tDCS to people with severe stroke, participants were excluded if they were able to actively extend the wrist more than 10°, extend 2 or more digits more than 10°, and abduct the thumb more than 10° (*n* = 7) [32]. Patients with difficulty to follow the procedures or understand the instructions (*n* = 2), cognitive deficits (*n* = 3), or other contraindications for tDCS such as pacemaker (*n* = 4) and metal in the head (*n* = 2) were excluded [3]. A total of 18 patients did not meet the above criteria and were excluded from the study.

### 2.3. Randomization

**Figure 1:**
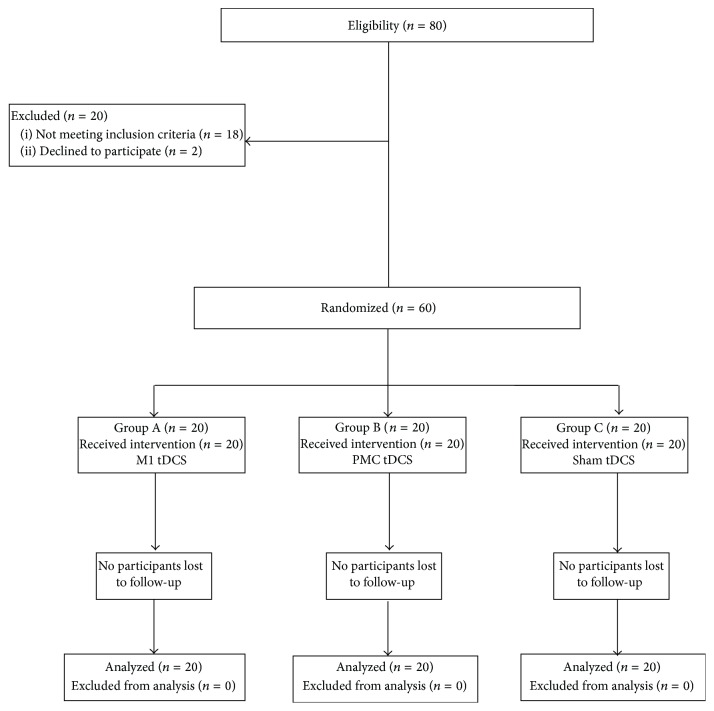
Flowchart of study based on Consolidated Standards of Reporting Trials (CONSORT).

In Figure 1, the CONSORT (Consolidated Standards of Reporting Trial) flow chart shows the number and distribution of participants. Patients were randomized to 3 groups, with twenty participants each: Group A, anodal stimulation in ipsilesional M1 and CIMT; Group B, anodal stimulation in ipsilesional PMC and CIMT; Group C, sham stimulation and CIMT.

The method of randomization was a 1 : 1 : 1 permuted block randomization generated by a web based randomization tool (https://www.random.org). This sequence was done independently and remotely by a blinded investigator who had no contact with other research procedures. After the randomization process, a blind researcher (not involved with the recruitment, data collection, or intervention) conducted the allocation of participants between the groups. This was employed by concealed allocation of sequentially numbered, opaque sealed envelopes, so that the person responsible for allocation had no contact with patients or with the work of others. This envelope was delivered one day before the treatment sessions to the researcher responsible for neurostimulation and the staff responsible for the execution of CIMT who were not involved with the other procedures. Data analysis was conducted by a researcher not involved in any stage of recruitment, screening, assessment, or intervention.

### 2.4. Outcome Measures

An initial neurological evaluation involving demographic and clinical data such as age, gender, lesion site, stroke time, and functional impairment (National Institute of Health Stroke Scale) was performed. The outcomes of the study evaluated functional independence, motor recovery, spasticity, gross motor function, and muscle strength of the affected side.

Functional assessment was made by the Barthel Index (BI), which analyzes the level of functional independence of patients for the ability to perform ten basic activities of daily living (range 0–100) [33].

Fugl-Meyer Assessment-Upper Extremity (FM) scale was performed (score ranges from 0 to 66) to assess arm motor recovery. The FM offers impressive test-retest reliability (total = 0.98 to 0.99; subtest = 0.87 to 1.00) [34].

Modified Ashworth Scale (MAS) was implemented for evaluating muscle tone in the shoulder adductors, the flexors of the elbow, wrist, fingers, and the thumb. The MAS score (0–5) of all muscles was summated to get a total MAS score that ranged from 0 to 25 [35].

Box and Block Test (BBT) was used to evaluate gross motor function. It counts the number of blocks that can be transported from one compartment of a box to another within 1 minute [36].

Medical Research Council (MRC) scale was used to record muscle power in shoulder abductors, flexors, and extensors of the elbow, the wrist, the fingers, and the thumb. Each of the above muscle groups was scored with degrees ranging from 0 (no motion is observed) to 5 (normal force against the total resistance). The total MRC score ranging from 0 to 45 included a proximal (MRC-prox, 0 to 15) and a distal subscore (MRC-dist, 0 to 30) [37].

A tDCS side effects questionnaire (headache, neck pain, burning, redness, and/or itching) was administered after each session. All assessments in this study were carried out by a trained researcher. Participants received standard instructions and were also closely supervised by the assessor during all tests.

### 2.5. Intervention

#### 2.5.1. tDCS

Each patient received 10 sessions (5 consecutive days for 2 weeks) of anodal tDCS or sham stimulation, with an intensity of 0.7 mA. For those who received sham stimulation, there was an emission current for only 30 seconds, a blinding method considered reliable for several previous studies [2, 12–14]. The current was delivered through 2 saline-soaked sponge surface electrodes using a battery-driven constant current stimulator (Trans Cranial Technologies®, Hong Kong).

We used a specific active electrode (6.4 × 2.5 cm) to avoid covering of adjacent areas by the tDCS electrode. We adopted the methods suggested by Schmidt et al. [38] and used a stimulation electrode with a smaller surface area (16 cm^2^ versus conventional 35 cm^2^), a rectangular instead of a square shape, and reduced total current (0.7 mA versus conventional 1.0 mA).

**Figure 2:**
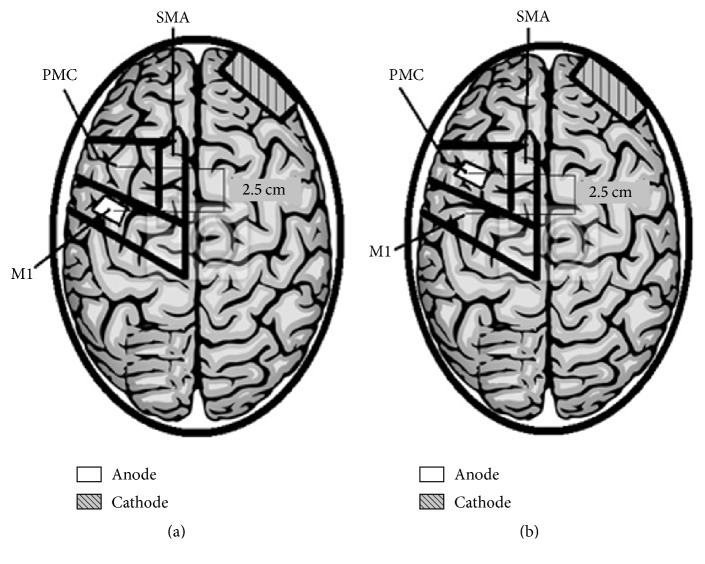
Electrode montage of 10 tDCS sessions. (a) M1 group. (b) PMC group. SMA: Supplementary Motor Area. Figure adapted from Pavlova et al. [2].

To stimulate the M1, the active electrode was placed over C3 or C4, according to the international 10–20 EEG system [39]. The PMC was defined as being the 2.5 cm anterior to the M1 motor area [40]. The anode was placed on the affected hemisphere and the reference electrode (with a size of 35 cm^2^) on the supraorbital region in the contralateral hemisphere. In addition to the 10 × 20 EEG system, we used the conversion of the Talairach coordinates into real space for the individual participants. Thus, magnetic resonance imaging of all participants was obtained before the experiment. The FSL software (FMRIB's Software Library, University of Oxford, UK) was used to transform coordinates for PMC and M1 for each subject individually. The individual structural images were initially converted into MNI coordinates (standard Montreal Neurological Institute) and then the MNI coordinates were inversely transformed to the original imaging space. MNI coordinates for M1 were (*x*  =   − 39, *y*  =   − 20, *z*  =  55), and for PMC they corresponded to (*x*  =   − 42, *y*  =   − 14, *z*  =  50). These values were obtained by converting Talairach coordinates to the MNI space [41]. These coordinates were then used to guide the frameless stereotaxy (Brainsight system, Rogue Research, Montreal, Canada). The location of target regions was performed by an experienced and trained professional. Procedures were repeated at each treatment session for all participants involved.  Figure 2 shows the electrode placement of 10 tDCS sessions for M1 and PMC group.

#### 2.5.2. CIMT

The CIMT was performed immediately after the neurostimulation session on a 3-hour daily protocol of motor skills training for two weeks (10 days, excluding weekends), with the supervision of a physiotherapist who was blinded to the other procedures. There was restriction of the unaffected upper extremity for 90% of waking hours of the patients, who were encouraged to use the affected limb during their daily activities [42].

### 2.6. Data Analysis

Statistical analyses were conducted using the Statistical Package for Social Sciences (SPSS, version 20.0). We used the intention-to-treat principle, with inclusion of all participants who attended at least one of the intervention sessions. We compared baseline characteristics using Chi-square test for categorical variables and Wilcoxon rank sum for continuous variables.

For the clinical outcomes, we made an a priori decision to use nonparametric statistics due to unevenly distributed data set. We assessed between-group differences (M1, PMC, and sham) using Kruskal-Wallis test, with post hoc Mann–Whitney* U* test/Dunn-Sidak adjustment, and within-group change (pre to post) using Wilcoxon signed rank test. Further, we determined the overall effect size using Cliff's delta test. All tests were 2-tailed and differences were considered statistically significant at *p* < 0.05.

## 3. Results

All participants completed the sessions, with no losses throughout the study, as shown in Figure 1. There were no significant differences in relation to the characteristics of the participants at baseline (*p* > 0.05) (Table 1).

### 3.1. Primary Outcome

**Figure 3:**
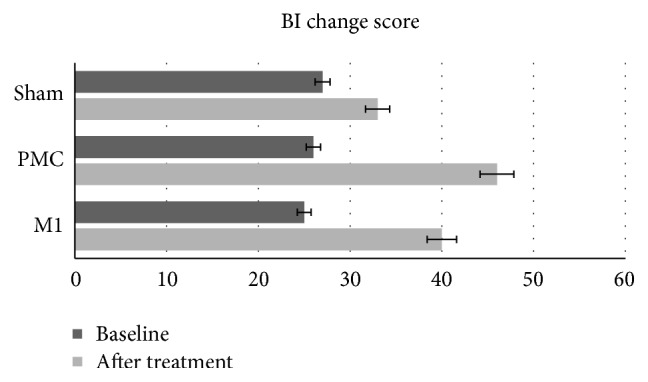
Comparisons of tDCS effects and CIMT on functional independence.

With regard to functional independence, the results are shown in Figure 3. Wilcoxon signed rank test showed significant difference from baseline to posttreatment for all groups (PMC: *z* = −2.11, *p* = 0.01; M1: *z* = −1.94, *p* = 0.01; sham: *z* = −1.85, *p* = 0.01). The improvement in the PMC and M1 groups had a large effect size (*δ* = −0.83, *p* = 0.02; *δ* = −0.64, and *p* = 0.01, resp.), where the effect size of the sham group was small (*δ* = −0.14, *p* = 0.01).

Kruskal-Wallis tests showed significant difference between groups (*χ*^2^ = 11.41, d.f. = 2, and *p* = 0.01). Post hoc comparisons indicated that there was a significant difference between PMC-M1 (*z* = −2.04, *p* = 0.03), PMC-sham (*z* = −2.78, *p* = 0.04), and M1-sham groups (*z* = −2.27, *p* = 0.02) at the end of intervention.

Subsequent analyses were conducted using models adjusted for variables indexing baseline functional characteristics since these variables have an influence on final performance. However, a significant difference in baseline BI does not exist (*p* = 0.82). When the patients are matched according to their baseline BI every patient in the PMC group had a greater percent increase in BI when compared to their matched pair in the M1 (*p* = 0.04) and sham groups (*p* = 0.02).

### 3.2. Secondary Outcomes

**Figure 4:**
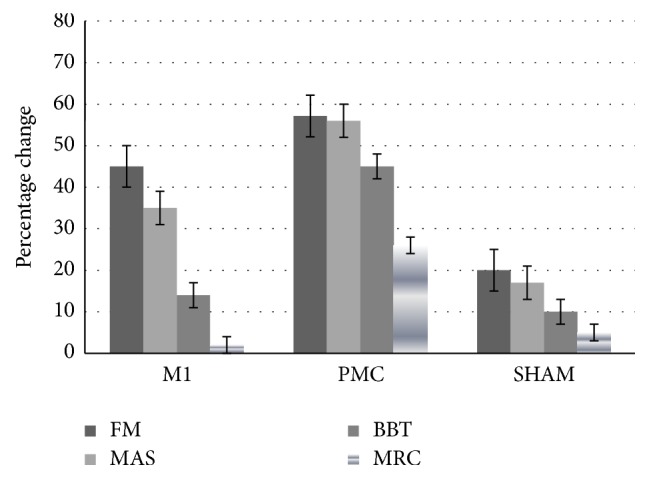
Percentage change in secondary outcomes from baseline to endpoint in the experimental groups. FM: Fugl-Meyer Assessment-Upper Extremity scale. MAS: Modified Ashworth Scale. BBT: Box and Block Test. MRC: Medical Research Council scale.

Results of secondary outcomes are reported in Figure 4. We found a significant difference between groups after treatment. Results of post hoc contrast analyses showed that the PMC group improved more on the motor recovery (FM score) than did the M1 (*z* = −2.01; *p* = 0.04) and sham groups (*z* = −2.36; *p* = 0.03). The spasticity (MAS score) decreased by 9 points in PMC group versus 6 points in M1 (*z* = −2.12; *p* = 0.02) and 3 points in sham group (*z* = −2.51; *p* = 0.04). Gross motor function (BBT score) and muscle power (MRC score) were improved only in PMC group (*p* < 0.04).

Although the results showed that the three groups had a better performance in the secondary outcomes over the course of the trial (Table 2), only PMC and M1 group showed large effects in motor recovery and spasticity (Cliff's delta > 0.60). The sham group showed small effects in all measures (when comparing baseline versus end of intervention).

### 3.3. Adverse Effects

16 out of 60 patients reported mild side effects after stimulation (7 in the M1 group, 6 in PMC group, and 3 in the sham group): skin redness under the site of stimulation (5 in M1 group, 4 in PMC group, and 3 in sham group), mild headache (3 in M1 group and 2 in PMC group), and sleepiness (1 in PMC group). In all groups some subjects experienced multiple adverse effects.

## 4. Discussion

In this work, we demonstrate that the application of PMC tDCS and M1 tDCS combined with CIMT results in significant motor improvement, higher than the CIMT applied alone with sham current. Our findings indicate that PMC stimulation has superiority over M1, supporting the notion that this region can be an alternative locus during poststroke rehabilitation.

In our experiments, PMC tDCS had a specific influence on motor function compared to M1 tDCS in all evaluated clinical outcomes. Consistent with our findings, Cunningham et al. [21] tested the effects of tDCS on ipsilesional higher motor areas paired to CIMT. The authors found gains in function and dexterity, as measured by the Upper Extremity Fugl-Meyer test, Nine-hole peg test, and Motor Activity Log, only for patients who received active stimulation. The authors state that ipsilesional PMC can help recruit the contralesional hemisphere as an adaptive role when severely impacted patients are involved.

Plow et al. [43] attributed this PMC performance to the ability to remap its organization, since its multilayer structure contains abstract and discrete somatotopic organization, allowing it to be an efficient substitute in response to the injury in the primary sensorimotor cortex. The PMC and M1 have a wide network of connections, with inputs from parietofrontal networks and projections for spinal cord that innervate the proximal and distal muscles [13]. Such connections could be useful if they are paired with a therapeutic protocol with the neurophysiological basis of CIMT, which aims to stimulate the paretic side with concomitant reduction of hyperactivity in the unaffected hemisphere, through the interhemispheric inhibition mechanisms [32].

In a previous study that sought to analyze potential reorganization in the area of hand representation corresponding to the PMC, Frost et al. [44] observed that the greater the damage in M1 caused by stroke, the greater the neural plasticity in the adjacent motor pathways, since an increase in hand representation in PMC was observed with values above 30% after vascular injury in M1. Along this perspective, Stewart et al. [45] evaluated the role of the premotor cortex in relation to motor action planning in poststroke individuals. The researchers observed that changes in the premotor component may negatively impact performance and learning of complex movements, suggesting that PMC should be a priority target for rehabilitation protocols that seek to improve the function of poststroke residual cerebral circuits.

An important aspect to be observed in our study is that we noticed a pattern of improvement in all groups, including the sham group (although it was lower than the other), comparing their baseline with final scores. This performance is probably due to the effectiveness promoted by the CIMT treatment protocol, as evidenced by other studies with poststroke patients [46, 47]. The mechanism by which the CIMT leads to neuroplasticity may involve the formation of various anatomical connections through neuronal sprouting, increasing the effectiveness of existing synaptic connections or even the recruitment of large numbers of neurons in the innervation of the adjacent extremity of those involved before the lesion [48].

The results of this preliminary study should be interpreted with caution, given some limitations. Although our goal has been to compare the efficacy of tDCS in PMC and M1 after stroke, we cannot guarantee that there has not been a cumulative effect of PMC stimulation reaching M1 and vice versa. Due to the existence of intra- and interindividual variations that interfere with the effects of neurostimulation [49], similar studies draw attention to this limitation related to the focal power of the technique, so that a current propagation effect from one region to another cannot be definitively excluded [21, 40]. However, it is important to note that we have used several strategies to locate PMC and M1 in distinct ways, such as reduced electrode size, use of standardized coordinates in previous studies for localization of target regions, use of MRI applied individually for each patient, and procedural repetition at each stimulation session. However, future studies should take into account variations in current density according to individual differences [50]. Another point that should be considered is that our design did not include neurophysiological measures; thus we cannot ascertain the causal relationship between the integrity of the CST and improvement in the patients' functionality, according to the stimulated area. Considering that the patients included in this study did not present significant variations in relation to the clinical parameters at baseline, the different benefits achieved by the studied groups suggest that the differences are due to manipulation of the experimental variable, that is, the locus of stimulation, as the sample was homogeneous. However, we reinforce that it is not possible to measure the role of cortical excitability on the efficacy of tDCS in both PMC and M1 in this pilot study. The improvement achieved by the stimulation of alternative areas as verified in the present study can contribute to the design of clinical trials with larger samples and the use of more specific techniques such as diffusion tensor imaging and biomarkers.

## 5. Conclusions

The stimulation of the PMC or M1 combined with CIMT promotes recovery after stroke, with better performance on motor tests by patients who received anodal tDCS in PMC. This finding should be useful for future studies that wish to investigate the relationship of the PMC with other areas of the motor function but also has clinical importance since tDCS is a safe, inexpensive, and effective technique when applied in conjunction with CIMT.

## Figures and Tables

**Table 1:** Clinical and sociodemographic characteristics of participants at baseline.

	M1 (*n* = 20)	PMC (*n* = 20)	Sham (*n* = 20)	*p* value
Age, mean (SD), *y*	55.18 (4.21)	52.97 (3.19)	54.76 (4.28)	0.41
Gender (male/female), *n*	13/7	9/11	12/8	0.94
Ischemic/hemorrhagic lesion, *n*	14/6	11/9	15/5	0.63
Cortical/subcortical stroke, *n*	8/12	9/11	8/12	0.87
* Basal ganglia*	2	1	3	0.85
* PLIC-Thalamus*	3	1	0	0.53
* PLIC-corona radiata*	5	7	7	0.92
* Thalamus*	0	1	1	0.89
* Striatum*	2	1	1	0.85
* Cortical*	8	9	8	0.83
Volume size, mean (SD), cm^3^	17.8 (5.49)	23.1 (3.96)	22.7 (3.09)	0.67
Side of brain lesion (right/left), *n*	9/11	11/9	10/10	0.73
Months after stroke, mean (SD)	1.78 (1.75)	1.86 (1.52)	1.92 (1.36)	0.82
NIHSS score, mean (SD)	17.2 (0.4)	17.4 (0.9)	16.7 (1.3)	0.91

NIHSS: The National Institutes of Health Stroke Scale.

PLIC: Posterior limb of the internal capsule.

**Table 2:** Descriptive and inferential statistics of the secondary outcomes measures.

	Before test	After test	Within-group effects^b^	*δ*
*FM*				
M1 + CIMT	20 (16–24)	29 (24–31)	−2.04 (0.01)^*∗*^	−0.67^c^
PMC + CIMT	21 (18–23)	33 (29–35)	−2.86 (0.02)^*∗*^	−0.82^c^
Sham + CIMT	20 (18–22)	24 (21–26)	−0.44 (0.01)^*∗*^	−0.11
Between-group effects^a^	1.98 (0.77)	11.6 (0.02)^*∗*^		

*MAS*				
M1 + CIMT	17 (11–19)	11 (9–14)	−1.94 (0.01)^*∗*^	0.61^c^
PMC + CIMT	16 (12–18)	7 (5–10)	−2.87 (0.02)^*∗*^	0.71^c^
Sham + CIMT	18 (11–21)	15 (12–18)	−1.16 (0.03)^*∗*^	0.09
Between-group effects^a^	2.13 (0.82)	15.2 (0.03)^*∗*^		

*BBT*				
M1 + CIMT	1 (1–3)	3 (1–5)	−0.81 (0.73)	−0.14
PMC + CIMT	1 (1–3)	7 (4–9)	−2.98 (0.02)^*∗*^	−0.67^c^
Sham + CIMT	1 (1–4)	2 (1–4)	−0.31 (0.58)	−0.04
Between-group effects^a^	1.44 (0.60)	12.7 (0.01)^*∗*^		

*MRC*				
M1 + CIMT	4 (1–7)	5 (1–7)	−0.61 (0.52)	−0.10
PMC + CIMT	3 (1–7)	11 (8–14)	−3.01 (0.03)^*∗*^	−0.87^c^
Sham + CIMT	4 (2–7)	6 (2–8)	−0.89 (0.80)	−0.06
Between-group effects^a^	2.57 (0.97)	13.1 (0.02)^*∗*^		

Data are expressed as the median (interquartile range-IQR).

FM: Fugl-Meyer Assessment-Upper Extremity scale.

MAS: Modified Ashworth Scale.

BBT: Box and Block Test.

MRC: Medical Research Council scale.

*δ*: Cliff's delta score.

^a^Kruskal-Wallis test (*p* value).

^b^Wilcoxon test (*p* value).

^c^Large  effect  size.

^*∗*^ *p* < 0.05.
